# Delusion-proneness predicts COVID-19 vaccination behavior

**DOI:** 10.3389/fpsyt.2024.1450429

**Published:** 2024-11-25

**Authors:** Kasim Acar, Ariadni Karagiannidou, Andreas Olsson, Jan-Willem van Prooijen, Leonie J. T. Balter, John Axelsson, Martin Ingvar, Alexander V. Lebedev, Predrag Petrovic

**Affiliations:** ^1^ Centre for Psychiatry Research (CPF), Department of Clinical Neuroscience, Karolinska Institutet, Stockholm, Sweden; ^2^ Center for Cognitive and Computational Neuroscience (CCNP), Department of Clinical Neuroscience, Karolinska Institutet, Stockholm, Sweden; ^3^ Division of Psychology, Department of Clinical Neuroscience, Karolinska Institutet, Stockholm, Sweden; ^4^ Department of Experimental and Applied Psychology, Vrije Universiteit Amsterdam, Amsterdam, Netherlands; ^5^ The Netherlands Institute for the Study of Crime and Law Enforcement (NSCR), Amsterdam, Netherlands; ^6^ Department of Criminal Law and Criminology, Maastricht University, Maastricht, Netherlands; ^7^ Department of Psychology, Stress Research Institute, Stockholm University, Stockholm, Sweden; ^8^ Department of Psychiatry, Radboud University Nijmegen Medical Centre, Nijmegen, Netherlands; ^9^ Donders Institute for Brain, Cognition and Behaviour, Radboud University Nijmegen, Nijmegen, Netherlands; ^10^ Divison of Neuro, Department of Clinical Neuroscience, Karolinska Institutet, Stockholm, Sweden

**Keywords:** delusion proneness, psychosis, schizophrenia, conspiracy ideation, vaccination, COVID-19

## Abstract

**Introduction:**

Vaccination-related conspiracy ideation is related to reduced compliance with public health advice globally. Such beliefs have previously been linked to the delusion-proneness trait. However, it is not known how this extends to getting vaccinated.

**Methods:**

Here, we examined how delusion-proneness, as assessed by Peters et al. Delusions Inventory (PDI), is associated with COVID-19 vaccination in a sample of 273 subjects. We also examined whether delusion-proneness predicted the time to get vaccinated, after the vaccine became available.

**Results:**

Unvaccinated subjects were more delusion-prone than vaccinated subjects (W=2225.5, *p*<0.001, *effect-size*=0.27). Among vaccinated subjects, higher delusion-proneness was related to longer time to get vaccinated ( ${\text{r}}_{\text{s}}$ =0.27, *p*<0.001). These effects remained after adjusting for anxiety, ADHD, and ASD (Autism Spectrum Disorder) traits as well as for psychiatric diagnoses and sex. Path analyses indicated that the effect of delusion-proneness on vaccination rate was strongly mediated through COVID-19 conspiracy ideation, suggesting that delusion prone individuals first develop specific delusion-like ideas regarding vaccination, which then delays vaccination. An exploratory analysis of written text by subjects instructed to explain why they had vaccinated or not, revealed a difference in reasoning between the groups. Unvaccinated individuals were primarily motivated by concerns about personal safety and potential side effects, while vaccinated individuals stated a desire to protect themselves and others as the primary reasons to get vaccinated.

**Discussion:**

Our results suggest that delusion-proneness is a key factor for attaining conspiracy beliefs, at least in relation to COVID-19 pandemic, and associated with lower vaccination rates as well as longer time to get vaccinated.

## 1 Introduction

Vaccination-related conspiracy ideation can reduce an individual’s likelihood to comply with public health campaigns, which aim to reduce interpersonal transmission of COVID-19 (1, 2). Studies have shown that misinformation and conspiracy ideation impact vaccination intentions negatively (3, 4). Despite implemented efforts, approximately 25% of the EU population has not been vaccinated for COVID-19 as of today (5). The causes to why people avoid getting vaccinated are poorly understood, even though a role of certain psychological characteristics and personality traits have been suggested to be related to vaccination hesitancy (6–8).

Research suggests that individuals scoring higher on delusion-proneness, a personality trait in the normal population associated with overvalued and delusion-like beliefs (9, 10), are more likely to develop conspiracy beliefs related to the COVID-19 pandemic and the associated vaccination program (7, 11, 12). While several of these studies were cross sectional, delusion proneness was measured before the out-break of the COVID-19 pandemic in one study (7), suggesting a possible causal link. In addition to the delusion-proneness personality trait, cognitive biases associated with delusional ideation such as Bias Against Disconfirmatory Evidence (BADE) and Jumping to Conclusions (JC) may also play a role in the development of COVID-19 conspiracy beliefs (7, 11). Finally, conjunction fallacy, i.e. biases in probabilistic reasoning whereby people overestimate the likelihood of co-occurring events, have been suggested to be related to conspiracy ideation, including COVID-19 conspiracy ideation (13, 14). Thus, it is already well-established that delusion proneness and related style of information processing is associated with development of COVID-19 conspiracy ideas. However, it is not known how development of such conspiracy ideas affects vaccination status, which is the main aim of the present study.

While the delusion-proneness trait may be a driving factor for not getting vaccinated, there are potentially other psychiatric traits that may have a negative impact on vaccination rates (15, 16). For example, anxiety may influence the willingness to get vaccinated in several ways. It is possible that anxious individuals are more likely to get vaccinated due to worry about the complications related to COVID-19, but such individuals may also choose not to get vaccinated due to concerns about the possible adverse effects of the vaccination. To date, it is not clearly described which of these two behavioral pathways are more likely although anxiety has been shown to be associated with a more positive attitude toward vaccination in one study (17). On the other hand, anxiety has also been shown to drive conspiracy beliefs generally (18), as well as COVID-19 conspiracies specifically (19). Other studies have also found that external threats (such as the COVID-19 pandemic) trigger the behavioral inhibition system (BIS), associated with heightened vigilance and anxiety, which in turn activates distal defenses such as increased beliefs in conspiracy theories (20). Apart from trait-anxiety, several other psychiatric traits may impact vaccination behavior. For example, as there is a link between both Attention Deficit Hyperactivity Disorder (ADHD) and Autism Spectrum Disorder (ASD) traits with delusion-proneness (21), they may also impact decisions making related to vaccination. In line with this reasoning, previous evidence suggest that ASD traits are associated with increased conspiracy ideation (22). As ADHD- and ASD-traits are closely associated to delusion-proneness (21) and previous studies have suggested that anxiety may impact vaccination behavior, adjusting for them is of importance in order to understand whether delusion-proneness specifically relates to not getting vaccinated. Measuring these traits would also give a better insight into which psychiatric traits drive choice to vaccinate, apart from delusion-proneness and how much each trait contributes to this choice.

In summary, the general association between delusion-proneness and COVID-19 conspiracy ideas is already established (7, 11, 12). In the present study, we went one step further and tested whether delusion-proneness also is specifically linked to the vaccination rate both in terms of the outcome (vaccinated or not) and how long it takes to get vaccinated from when the vaccination program started. Also, we implemented a path analysis, in order to better describe whether the delusion proneness may be related to vaccination behavior through development of COVID-19 conspiracy ideas. Finally, in order to better understand the motives for not choosing (or choosing) to vaccinate we also performed an exploratory analysis focusing on how the subjects reasoned about their choice using a text analytic tool.

## 2 Materials and methods

### 2.1 Subjects and general study design

**Figure 1:**
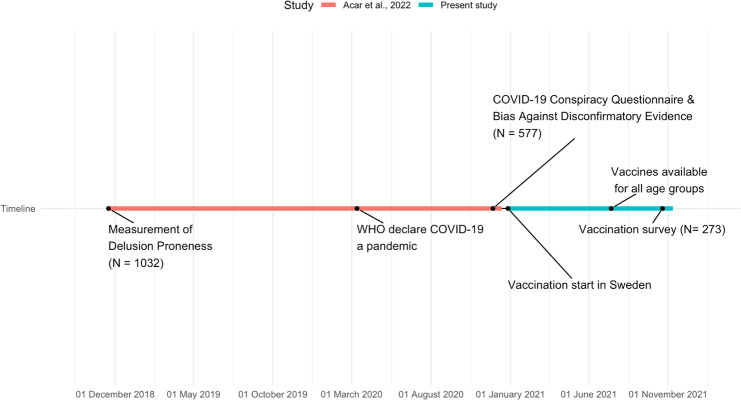
Timeline showing dates of data sampling for the study where the relation between delusion proneness and later development of COVID-19 conspiracy believes was studied (7) and present study, including important dates pertaining to the COVID-19 pandemic.

We invited subjects from our previous studies on delusion-proneness and conspiracy ideation (7, 23) to complete a follow-up survey which consisted of questions pertaining to whether or not they have been vaccinated against COVID-19, the date for when the first dose was received, as well as to freely express in text why they chose to vaccinate or not. They also completed the State-Trait Anxiety Inventory (STAI-T) to assess their trait anxiety level (24). Previously, they had completed the Ritvo Autism Asperger Diagnostic Scale–Revised (RAADS) (25) to assess ASD traits and the adult ADHD Self-Report Scale (ASRS) (26) to assess ADHD-traits. The subjects were invited to complete the follow-up survey via email which was sent out in October 2021. Out of the 1032 invited subjects, 273 completed the follow-up survey. See **Supplementary Materials** for further details on the sampling procedure and **Figure 1** for a timeline of the current and previous study on the relation between delusion proneness and development of conspiracy ideation (7). Note, that the initial study from where we invited subjects was over-sampled for delusion proneness and use of psychedelics (23). Thus, the present population does not represent a general population in terms of delusion proneness distribution.

The range of age for the whole sample was 18-67 years (*N* = 273, *M* = 29.6, *SD* = 7.21), education (years of University studies or equivalent) was 0-12 years (*M* = 3.37, *SD* = 2.50), and mean score for PDI was 3.93 (*SD* = 2.99, *N* = 268). 197 (73.5%) of the 273 who answered the follow-up survey were female, whereas 71 (26.5%) were male. 36 (13.19% had not been vaccinated against COVID-19, and 237 (86.81%) had received a vaccine. 189 (70.5%) had not been diagnosed with a psychiatric disorder (major depressive disorder, psychotic disorder, obsessive compulsive disorder, ASD, ADHD, post-traumatic stress disorder and anxiety disorder), while 79 (29.5%) had been diagnosed with a psychiatric disorder. See **Table 1** below for more detail.

**Table 1:** Showing descriptive data of our sample (N = 273).

Characteristic	Vaccinated, N = 237^1^	Unvaccinated, N = 36^1^	Total N = 273^1^
Age			
Min - Max	18 - 67	21 - 47	18 - 67
Mean (SD)	30 (7)	30 (6)	30 (7)
Median [Q1 - Q3]	29 [25 - 33]	29 [26 - 34]	29 [25 - 33]
Sex			
Female	181 (76%)	21 (58%)	202 (74%)
Male	56 (24%)	15 (42%)	71 (26%)
PDI			
Min - Max	0 - 12	0 - 13	0 - 13
Mean (SD)	4 (3)	6 (3)	4 (3)
Median [Q1 - Q3]	3 [1 - 5]	6 [4 - 8]	3 [2 - 6]
Education			
Min - Max	0.00 - 15.00	0.00 - 10.00	0.00 - 15.00
Mean (SD)	3.57 (2.60)	2.37 (2.40)	3.42 (2.60)
Median [Q1 - Q3]	3.25 [2.00 - 5.00]	2.00 [1.00 - 3.00]	3.00 [1.00 - 5.00]
Psychiatric Diagnosis	62 (27%)	17 (49%)	79 (29%)

^1^n (%).

The study was performed in accordance with the Declaration of Helsinki (27), and approved by the Swedish Ethical Review Authority. All subjects gave informed consent prior to answering the survey. The main outcome analyses were performed adhering to a preregistered protocol (https://osf.io/npb8q/wiki/home).

### 2.2 Quantification and statistical analysis

To test differences in delusion-proneness (using PDI) between vaccinated and non-vaccinated groups we first conducted a Wilcoxon test due to non-normally distributed data. Similarly, we conducted a Spearman’s correlation to test correlation between PDI and time to vaccination.

We then applied a series of logistic regression analyses to investigate the association between COVID-19 vaccination status (either received or not) and a set of predictor variables. Starting with a base model, we sequentially incorporated relevant covariates across different models, such as ASRS, RAADS, STAI-T, psychiatric diagnoses, sex, and education, to adjust for potential confounders. Each regression model yielded beta weights, standard errors, z-values, p-values, and odds ratios to interpret the relationship between predictors and the likelihood of getting vaccinated against COVID-19.

Subsequently, we conducted linear regression analyses, where the response variable was the time taken to receive the vaccine. Again, various models were designed by adding the different predictors and covariates to elucidate their influence on the time to vaccination. Beta coefficients, t-values, and p-values were computed to gauge the significance and strength of these relationships.

Finally, a path analysis was carried out to discern direct and mediated effects of the predictors on the decision to get vaccinated. The analysis specifically explored how PDI impacts the decision to vaccinate, both directly and through its effect on COVID-19 Conspiracy Questionnaire (CCQ) Total – (see **Supplementary Material**). All models were scrutinized using appropriate statistical criteria to ascertain their validity and reliability (see **Supplementary Material**).

### 2.3 Exploratory analyses

#### 2.3.1 Word cloud and text analysis

We performed text analysis of the open question on why or why not the subjects had taken the vaccine. We used the “bing” sentiment lexicon (28) in the *R* programming language (29). We then created a word cloud showing the most common positive and negative words used by each group. Furthermore, we classified each subject’s comments based on the overarching theme that were identified and created a bar-chart showing the average frequency of themes in each group.

## 3 Results

### 3.1 Group comparison and correlational analysis

**Figure 2:**
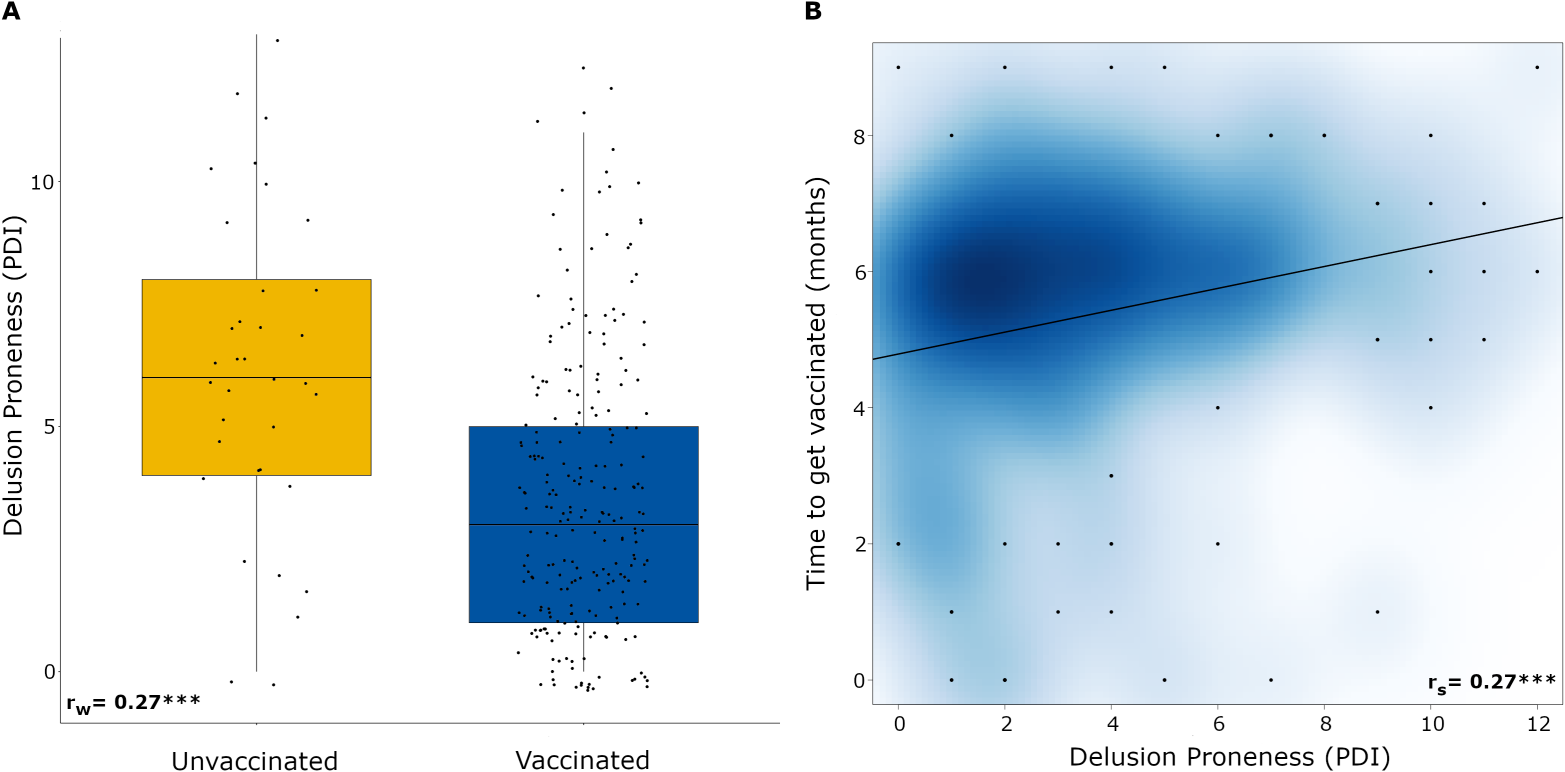
**(A)** Boxplot showing the comparison of means of PDI between unvaccinated and vaccinated subjects. Each data point represents an individual. **(B)** Scatter plot showing the relationship between PDI and time to get vaccinate (in months) from when the vaccination program started. The cloud represents accumulated effect of overlapping data points, while black dots represent single data points with jitter. The darker shade of blue represents more data points (*N =229)*, *** *p* <.001. PDI: Peters et al. Delusions Inventory. rw = Wilcoxon's effect size. rs = Spearman's correlation.

The first step of the analyses was focused on group comparisons (**Figure 2A**), which showed that unvaccinated individuals scored higher on delusion-proneness (*W*=2225.5, *p*<0.001, Wilcoxon's effect size =0.27). Notably, the scoring of delusion-proneness was performed before the launch of the vaccination program against COVID-19. For the subset of subjects that got vaccinated against COVID-19 (*N* = 237), we conducted a Spearman’s correlation which showed a positive correlation between PDI and time to get vaccinated (r_s_=0.27, p<.001), see **Figure 2B**.

### 3.2 Logistic regression

We performed a series of logistic regression models to analyze how well PDI predicted being vaccinated when potential confounders were added to the model. In all models a higher PDI predicted a reduced likelihood to vaccinate. The predictive power of PDI was only marginally affected when adding other psychiatric traits (Model 2), psychiatric diagnoses (Model 3), education and sex to the models (Model 4) which all are potential confounders (**Table 2**). Of interest, both psychiatric diagnosis and anxiety also predicted whether individuals got vaccinated, but in opposite ways (psychiatric diagnoses were associated with lower degree of vaccination, anxiety was associated with higher degree of vaccination). Testing for multicollinearity showed that minimal variance inflation factor remains for the logistic regression models (1.03-1.37), comparison of AIC of the logistic models showed that Model 4 fit the data better, see **Table 1** for more details.

**Table 2:** Likelihood to get vaccinated against COVID-19 (vaccinated vs non-vaccinated).

Characteristic	Model 1			Model 2			Model 3			Model 4		
	OR^1^	95% CI^1^	p-value	OR^1^	95% CI^1^	p-value	OR^1^	95% CI^1^	p-value	OR^1^	95% CI^1^	p-value
PDI	0.77	0.68, 0.87	**<0.001**	0.74	0.64, 0.84	**<0.001**	0.74	0.64, 0.84	**<0.001**	0.78	0.67, 0.89	**<0.001**
ASRS				1.02	0.98, 1.06	0.3	1.02	0.99, 1.07	0.2	1.02	0.98, 1.06	0.5
STAI-T				1.02	0.99, 1.06	0.2	1.04	1.00, 1.08	0.072	1.05	1.00, 1.09	**0.040**
RAADS-N				1.11	0.83, 1.54	0.5	1.10	0.82, 1.54	0.5	1.02	0.75, 1.43	>0.9
Psychiatric Diagnosis							0.31	0.13, 0.70	**0.005**	0.37	0.15, 0.90	**0.026**
Sex (M1F2)										1.78	0.74, 4.15	0.2
Education										1.17	1.00, 1.42	0.075
AIC	192			193			187			177		

^1^OR, Odds Ratio; CI, Confidence Interval.

A logistic regression model showed that higher PDI scores predicted a significantly reduced likelihood to get vaccinated when not adjusting for other variables (Model 1), and this effect remained significant when adjusting for psychiatric traits (Model 2), psychiatric diagnoses (Model 3) as well as sex and education that could mediate this effect (Model 4). ASRS, adult ADHD Self-Report Scale; PDI, Peters et al. Delusions Inventory; RAADS, Ritvo Autism Asperger Diagnostic Scale–Revised; STAI-T, the State-Trait Anxiety Inventory – Trait; *AIC, Akaike information criterion.*P-values set in boldface indicate statistical significance.

### 3.3 Linear regression

Next, four linear regression analyses were carried out to analyze what factors were associated with the time to get vaccinated (in months) in the group that had been vaccinated. For each model, predictors were added in a step-wise fashion in order to adjust for the effects of covariates. Similar to the results of the logistic regression for the binary outcome (vaccinated or not vaccinated), PDI was a stable predictor of the days it took to get vaccinated (**Table 3**). Of interest, both education and sex impacted how long time it took to getting vaccinated. While sex was associated with a longer time to get vaccinated, i.e. women took longer time to get vaccinated, education was associated with a somewhat quicker time to get vaccinated. Testing for multicollinearity showed that minimal variance inflation factor remains for the linear regression models (1.03-1.38), while comparison of AIC of the linear models showed that Model 4 had the best fit, see **Table 2** for further details on model comparison.

**Table 3:** Standardized linear effects of predictors on time to vaccination against COVID-19.

Characteristic	Model 1			Model 2			Model 3			Model 4		
	Beta	95% CI^1^	p-value	Beta	95% CI^1^	p-value	Beta	95% CI^1^	p-value	Beta	95% CI^1^	p-value
PDI	0.16	0.07, 0.25	**<0.001**	0.16	0.06, 0.25	**0.002**	0.15	0.06, 0.25	**0.002**	0.13	0.04, 0.23	**0.007**
ASRS				0.00	-0.02, 0.03	0.9	0.00	-0.02, 0.03	0.8	0.00	-0.02, 0.03	>0.9
STAI-T				0.00	-0.03, 0.02	>0.9	0.00	-0.02, 0.03	>0.9	0.00	-0.03, 0.02	0.9
RAADS-N				0.03	-0.16, 0.22	0.8	0.03	-0.16, 0.22	0.8	0.04	-0.14, 0.23	0.6
Psychiatric Diagnosis							-0.20	-0.80, 0.40	0.5	-0.37	-0.96, 0.23	0.2
Sex (M1F2)										0.67	0.07, 1.3	**0.027**
Education										-0.16	-0.26, -0.07	**0.001**
Adjusted R²	0.050			0.038			0.036			0.095		
AIC	949			954			956			919		

^1^CI, Confidence Interval.

A linear regression model showed that higher PDI predicted the number of days it took to get vaccinated when not adjusting for other variables (Model 1), and this effect remained significant when adjusting for psychiatric traits (Model 2), psychiatric diagnoses (Model 3) as well as sex and education that could mediate this effect (Model 4). ASRS, adult ADHD Self-Report Scale; PDI, Peters et al. Delusions Inventory; RAADS, Ritvo Autism Asperger Diagnostic Scale–Revised; STAI-T, the State-Trait Anxiety Inventory – Trait; *AIC, Akaike information criterion.*P-values set in boldface indicate statistical significance.

### 3.4 Adjustment for age

Adjusting for age was not straightforward as different age groups were offered to start vaccination at different periods, i.e. age was cofounded by possibility to get vaccinated. Therefore, we did not adjust for age in our main analyses, but treated age and time offered to vaccinate as noise in the data (adhering to the preregistration protocol). However, we also performed two sensitivity analyses to reduce the effect from these factors. We first conducted the same logistic and linear regression analyses but only with young adults between 18-35 years, who were offered vaccination at the same time, showing similar results. We also conducted the same logistic and linear regression analyses using all subjects where we adjusted for age. These analyses also showed similar results as the main analyses (see **Supplementary Tables S1–S4**). These results suggested that our main analyses were not confounded by age. As only 27 subjects were above 35 years, it was not possible to study whether different age groups differed in when they chose to get vaccinated.

### 3.5 Path analysis

**Figure 3:**
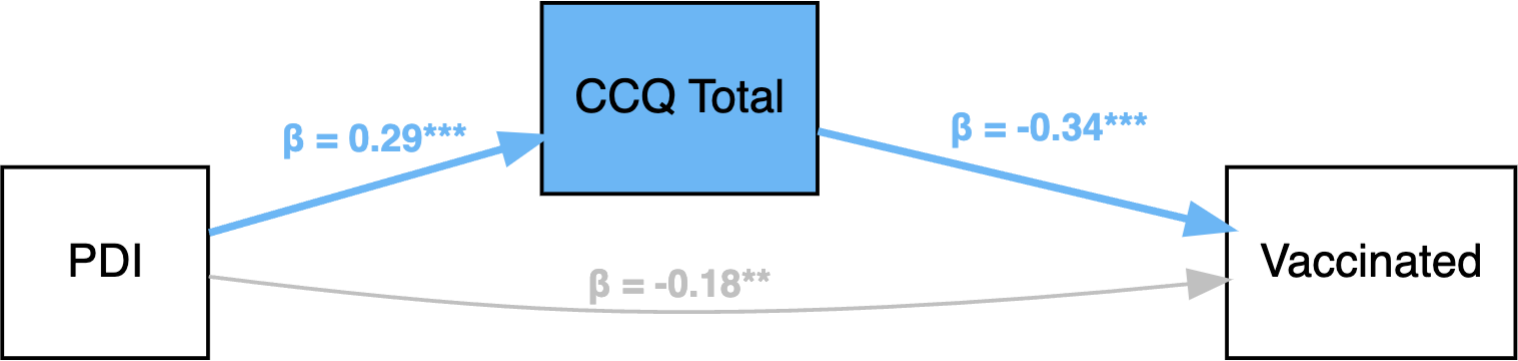
Path analyses (with standardized parameter values) of how delusion-proneness (measured with PDI) predicts vaccination with COVID-19 Conspiracy ideation as a mediator. PDI = Peters et al. Delusions Inventory; CCQ Total = total score on the COVID-19 Conspiracy Questionnaire. ** *p* <.01, *** *p* <.001.

A path analysis using *r* package (*lavaan)* with the Sobel test was performed to examine the mediating role of COVID-19 Conspiracy Questionnaire (CCQ)^6^ between PDI and vaccination against COVID-19. We found that CCQ partially mediates the relationship between PDI and vaccination (*β* = -0.18, *z* = -3.17, *p* = .002). See **Figure 3** and **Supplementary Materials** for details.

### 3.6 Exploratory analyses

#### 3.6.1 Interaction analysis

As we observed a main effect for both delusion-proneness and anxiety in our main analyses we examined the relationship between delusion-proneness, anxiety and vaccination against COVID-19, performing a logistic regression with an interaction term. The interaction showed that it was particularly individuals with both low anxiety and high delusion-proneness that were less likely to get vaccinated (N = 273, Z *= 3.588, p=0.003*) (**Supplementary Figure S1**).

### 3.7 Text analysis

**Figure 4:**
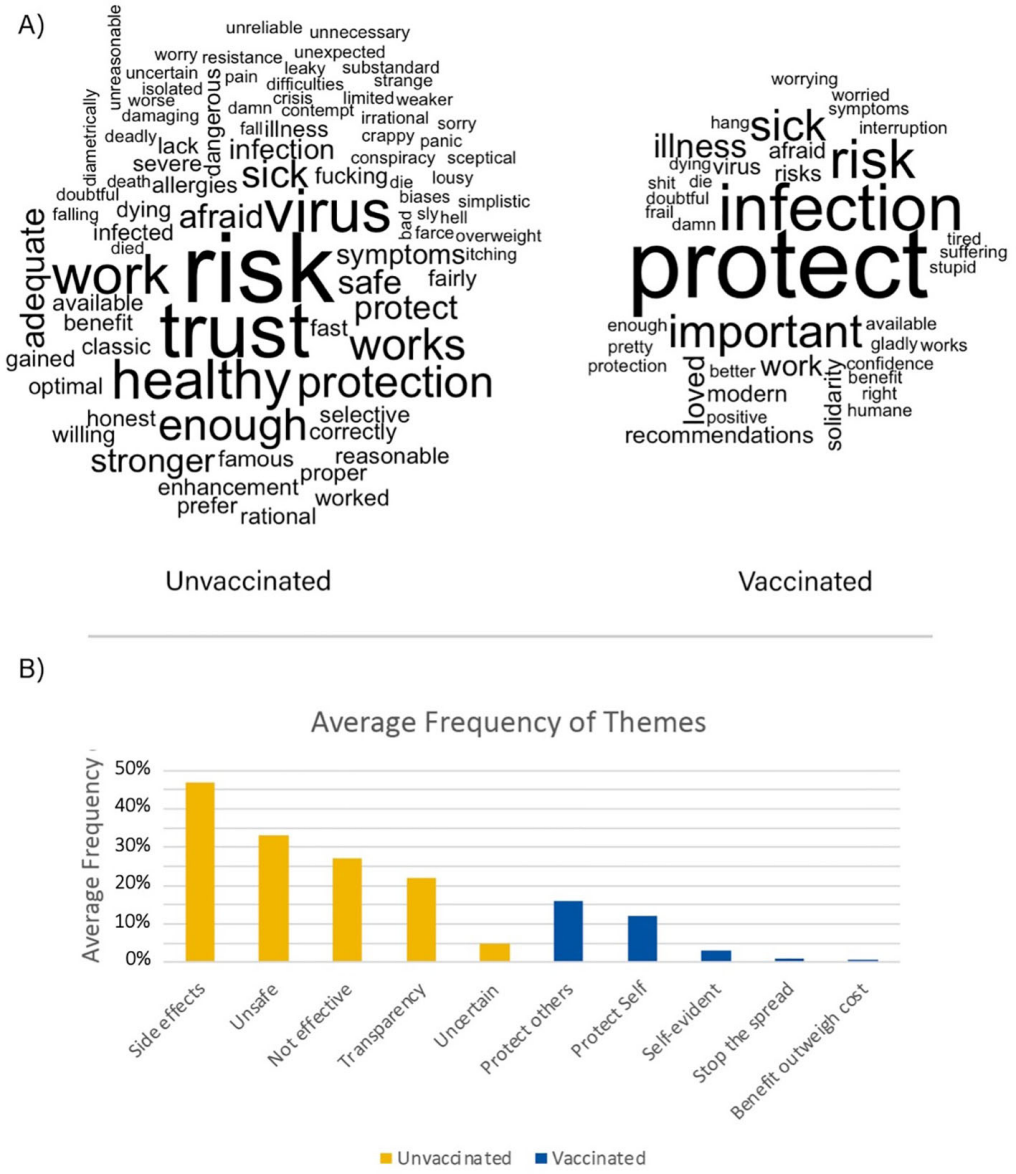
**(A)** Word cloud showing most frequent words used by unvaccinated subjects (left) and for vaccinated subjects (right). The bigger words are more frequently used than the smaller ones. Results from the text analysis suggested that unvaccinated individuals used more negative words and themes regarding the vaccination while vaccinated individuals used more positive words about protecting themselves and other individuals. Thus, while both groups were concerned about safety issues, conceptualizations of safety differed between them. A group comparison was performed in order to test if the average number of words used was significantly different between vaccinated and unvaccinated subjects and found that on average, unvaccinated subjects used more words (mean=52.03, SD=83.67, median=20.5, range 2-422) to describe why they did not get vaccinated compared to the vaccinated subjects (average=14.74, SD=10.92, median=11, range 2-61) (W=989.9, p=.002). This result remained significant even after excluding two extreme cases in the non-vaccinated group (W=989.5, p=.009). We then conducted a correlation analysis and found no significant correlation between number of words and PDI (r=-.22, p=0.22) or CCQ (r=-.09, p=0.62). **(B)** Average frequency of themes found in subject’s responses on why they did or did not get vaccinated (unvaccinated n = 36, vaccinated n = 237). Note that every subject’s response was tested for all identified themes.

Subjects were instructed to explain why they had taken a COVID-19 vaccine or not, in writing. The responses were text-analyzed to extract most commonly occurring words and sentiments from the full written text. Lastly, we classified every subject’s comment on why they did or did not get vaccinated against COVID-19 into themes (see **Figures 4A, B**).

## 4 Discussion

While several studies have suggested that conspiracy ideation relates to lower *intention* to get vaccinated (30–33), the present study expands on the previous results in showing that delusion-proneness and conspiracy ideation was linked to whether or not individuals also got vaccinated, as well as how long time it took to get vaccinated in the vaccinated group. Namely, individuals who were not vaccinated against COVID-19 scored significantly higher on the trait delusion-proneness measured by PDI. Additionally, we showed a positive correlation between PDI and time to get vaccinated in the vaccinated group. These effects were robust and remained after adjusting for demographic factors, psychiatric diagnoses, and psychiatric traits relating to anxiety, ASD, and ADHD as well as age.

It is important to consider confounds from other psychiatric traits as previous studies have shown a comorbidity between delusion-proneness, ADHD- and ASD traits (21). Our study suggests that specifically delusion-proneness is associated with the development of these conspiracy theories and ultimately a hesitancy against vaccination. Another trait identified as an important contributor to the outcome was anxiety, as anxiety has been previously associated with a more positive attitude toward vaccination (17), which is in line with our finding showing that subjects with high trait-anxiety are more likely to vaccinate against COVID-19. However, we did not find an effect of trait anxiety in our time analyses. Interestingly, we observed an interaction effect showing that subjects with low anxiety and high delusion-proneness were less likely to get vaccinated against COVID-19, compared to high anxiety and high delusion-prone individuals. ASD and ADHD-traits did not have any association with vaccination behavior in our study. Thus, to our knowledge our results are the first to show that delusion-proneness is a significant factor explaining some of the variance in vaccination behavior, and remains significant when controlling for other major psychiatric traits that suggest no negative impact on vaccination. In order to better understand the relative importance of delusion-proneness on vaccination behavior, future studies should include a broader range of personality traits and traits associated with psychiatric disorders.

It should be noted that delusion-proneness is a personality trait observed as a continuum in the population (10). While individuals exhibiting high levels of delusion-proneness may share certain cognitive biases with psychotic disorders (34–37), it is important to avoid pathologizing this personality trait that often is associated with a normal, and sometimes even favorable, functioning (38, 39).

The present project is longitudinal in that it follows the development of conspiracy ideation and their later consequences over a period of almost three years (**Figure 1**, **Supplementary Materials**). As we measured delusion proneness 2018 and 2019, before the COVID-19 pandemic, and then we followed the group into the pandemic, we could study how conspiracy beliefs were developed 2020 in relation to previously established delusion proneness and associated cognitive biases (7). The present study is based on the third measurement on this population, focusing on how delusion proneness and development of COVID-19 conspiracy beliefs is associated with later vaccination behavior (measured autumn 2021). A key question is why delusion proneness trait is related to development of conspiracy ideas that determines later behavior? Theoretical considerations have suggested that the psychosis phenotype is associated with an imbalance in the hierarchical predictive coding system of the brain with weak and unprecise low level priors, leading to development of multiple error signals (on an information processing level) and experience of aberrant salience (on a phenomenological level) (40, 41). Such aberrant salience calls for novel explanations that can incorporate them into a coherent explanation of the world, triggering the development of delusional ideas (40, 41). Thus, it may be suggested that development of conspiracy ideas is a consequence of the cognitive style of information processing associated with delusion-proneness. This mechanism suggests that a key component in decreasing vaccination hesitancy would be based on how information is given.

As it took longer to get vaccinated for those who scored high on delusion-proneness, such individuals may require more information before taking the final decision to vaccinate. This finding suggests that under the right circumstances, individuals with high delusion-proneness may accept vaccination. For example, individuals with high delusion-proneness may eventually be persuaded to get vaccinated when information is communicated through different channels, and addressing their safety concerns.

We also found that it took longer time for women to get vaccinated compared to men. This is in line with a recently published systematic review and meta-analysis (42), showing that women in the general population, especially women who work in healthcare have less intentions to get vaccinated against COVID-19 than men.

In the study, measurement of delusion-proneness was performed before the COVID-19 pandemic, whereas measurement of COVID-19 conspiracy ideation was performed during the pandemic, and the assessment of whether individuals had vaccinated or not was performed in a third step. Our path analysis supports that subjects with high delusion-proneness acquire COVID-19 conspiracy ideas more often than others, which increases the risk to avoid vaccinations. However, previous research suggests that the opposite is possible as well, i.e., intentions to not get vaccinated may in fact strengthen and develop conspiracy ideation (32). While such reverse causation is possible for the development of specific conspiracy ideas, it is likely that basic aspects of delusion-proneness predict how novel beliefs are acquired.

Our text analysis for the reasons the subjects got vaccinated or not, showed that unvaccinated subjects tended to use more negative words and themes regarding the vaccine, such as its effectiveness, lack of transparency as well as that it is unsafe and that they are afraid of the side effects. For vaccinated subjects, we observed more negative words and themes directed at the virus and the disease, whereas the positive words were related to protection of self and others, solidarity and that it is self-evident to take the vaccine to stop the spread. In line with this, unvaccinated individuals used more words in general, possibly suggesting a stronger desire to justify their decision. This could be related to that unvaccinated people reason why they *did not* get vaccinated, while vaccinated people reason why they *did* get vaccinated.

There are a number of limitations in the present study. Although the used metrics leveraged in the present study (scores on PDI) have been shown to have a good test-retest reliability (9, 26, 43) our study measures are self-rated, which limits the objectivity of the evaluations. Another limitation of the present study is that somatic diseases or other psychiatric and personality traits than the ones measured here, may have an impact on willingness to get vaccinated. Also, while we cannot exclude selection bias in this study, the distribution of PDI scores was not suggestive of selection bias within this dimension. However, as there was an oversampling of high delusion proneness in the initial study (23) from where we invited the subjects to participate in the present study, we cannot directly infer our results to a general population. Furthermore, education was only measured as numbers of years of university education (or similar) as a large majority of Sweden’s population complete secondary education. Finally, another limitation of our study is the small sample size which one needs to consider when interpreting the results presented in the paper. The main strength of the study is that it follows a cohort from before the COVID-19 pandemic, over the pandemic and until a vaccination has been available for everyone. This gives a unique opportunity to study formation of conspiracy beliefs and the later behavioral consequences.

In summary, our results show that individuals with higher levels of delusion-proneness are more reluctant to get vaccinated against COVID-19, and that this is not only driven by the general cognitive style of delusion-prone trait phenotype, but also by development of beliefs in COVID-19 conspiracy theories. Importantly, of the individuals with high delusion-proneness that vaccinated, it took longer time to get vaccinated after vaccines were available. On a more general level, the present findings suggest that a reluctance to get vaccinated may represent an example of how neurocognitive and psychiatric traits may impact real-life behaviors with direct implications for public health and society. Our results may also have implications and give input for vaccinations programs for children, where vaccine rates have been shown to be dropping (44). Future studies should aim to investigate how providing more tailored information and public campaigns, related to an individual’s vaccination and safety concerns, can be implemented to better support people in regard to their decision-making processes related to pandemics.

## Data Availability

The datasets presented in this study can be found in online repositories. The names of the repository/repositories and accession number(s) can be found below: https://github.com/kasimacar/CCQOnlineBADE.
