# Supplementary material for: Delusion-proneness predicts COVID-19 vaccination behavior
Source: Front Psychiatry. 2024 Nov 25;15:1450429. doi: 10.3389/fpsyt.2024.1450429 (PMC11625794; doi:10.3389/fpsyt.2024.1450429)
Supplement: Supplementary file 1 [file DataSheet1.docx]

**Supplements**

**Delusion-proneness predicts COVID-19 vaccination behavior**

Acar K^1,2^, Karagiannidou A^1,2^, Olsson A^2,3^, van Prooijen JW^4,5^, Balter LJT^3,7^, Axelsson J^2,3,7^, Ingvar M^1^, Lebedev AV^1,2^*, Petrovic P^1,2^*

^1^Centre for Psychiatry Research (CPF), Department of Clinical Neuroscience, Karolinska Institutet, Stockholm, Sweden

^2^Center for Cognitive and Computational Neuroscience (CCNP), Department of Clinical Neuroscience, Karolinska Institutet, Stockholm, Sweden

^3^Division of Psychology, Department of Clinical Neuroscience, Karolinska Institutet, Stockholm, Sweden

^4^Department of Experimental and Applied Psychology, Vrije Universiteit Amsterdam.

^5^The Netherlands Institute for the Study of Crime and Law Enforcement (NSCR), The Hague, The Netherlands.

^6^Department of Criminal Law and Criminology, Maastricht University.

^7^Stress Research Institute, Department of Psychology, Stockholm University, Stockholm, Sweden

^8^Divison of Neuro, Department of Clinical Neuroscience, Karolinska Institutet, Stockholm, Sweden

* = Contributed equally to the study

**Sample**

The present study is a part of a larger longitudinal project where we want to better understand behavior and cognition related to delusion proneness and use of psychedelics. In so far, we have published three studies based on this sample (Lebedev et al., 2021;Acar et al., 2022;Lebedev et al., 2023). A main research question is focused on how conspiracy ideas are formed and which consequences they have. For this research question we have published one study showing how delusion proneness at the start of the study (2018/2019) is associated with development of COVID-19 conspiracy ideas 2020 (Acar et al., 2022). The present study is focused on how these traits relate to vaccination behavior 2021 (see also Figure 1).

At the start of the project, we assessed delusion-proneness in a screening survey of an adult population before the outbreak of the COVID-19 pandemic (year 2018/2019; n=1032) (Lebedev, 2021). These subjects were included through web-based announcements on social media services, on forums which were expected to include our target populations (Lebedev, 2021). In the first gathering of data we included measures of delusion and psychosis proneness, recreational use of drugs including psychedelics, psychiatric diagnoses, socio-economic factors, Attention Deficit Hyperactivity Disorder (ADHD) and Autism Spectrum Disorder (ASD) traits and other variables pertaining to mental health (Lebedev, 2021). The measures that are used in the present study are further described in the present main manuscript. During the pandemic, this cohort underwent assessment of COVID-19 conspiracy ideation (2020; n=577 participated) (Acar et al., 2022). For that purpose, we constructed a conspiracy questionnaire focused on the COVID-19 pandemic (COVID-19 Conspiracy Questionnaire; CCQ) that measures common COVID-19 conspiracy-related statements (and which is used in the present study). Apart from rating the CCQ, a test measuring cognitive bias for inflexible believes was performed by the subjects (not used in the present study).

Following vaccination becoming available for all ages, we gathered information on vaccination behavior (see Survey Questions below) from all subjects enrolled from the start of the study (2021; n=273 participated). We also gathered information on general anxiety levels (see main manuscript). Of the sample from 273 subjects, 79 had a history of psychiatric diagnosis of which 3 had been diagnosed with schizophrenia, 23 with depression, 11 with bipolar disorder, 13 with ADHD, 10 with Autism, 7 with OCD, while 12 subjects also indicated “Other” which included PTSD, anxiety disorders and emotionally unstable personality disorder.

**Detailed Results of Logistic Regression Models**

We performed several logistic regression analyses to explore the relationship between COVID-19 Vaccination (yes or no), and our predictors. For Model 1, the standardized Beta weight for the Constant was (*N* = 267, *β* = 3.14, *SE* = 0.38, *z* = 8.18, p < .001). The standardized predictor variable was (*β* = -0.26, *SE* = 0.06, *z* = -4.34, *p* < .001), the estimated odds ratio showed a decrease of 23% (*Exp* (B) = 0.77, 95% CI [-0.38, -0.144] for Vaccination against COVID-19 for every one unit increase of PDI. In Model 2, we added ASRS, RAADS and STAI-T as covariates in order to control for the effects of these traits. For this model, the standardized Beta weight for the Constant was (*N* = 267, *β* = 1.88, *SE* = 0.81, *z* = 2.31, p = .02). The standardized beta weight for PDI was (*β* = -0.30, *SE* = 0.07, *z* = -4.46, *p* < .001), the estimated odds ratio showed a decrease of 26% (*Exp* (B) = 0.74, 95% CI [-0.44, -0.17] for Vaccination against COVID-19 for every unit of increase in PDI. ASRS (*β* = 0.018, *SE* = 0.019, *z* = 0.96, *p* = 0.33), STAI-T (*β* = 0.024, *SE* = 0.018, *z* = 1.27, *p* = .2), RAADSN (*β* = 0.1, *SE* = 0.16, *z* = 0.64, *p* = .51). In Model 3, we added psychiatric diagnoses as covariates. It showed that the standardized Beta weight for the Constant was (*N* = 267, *β* = 1.78, *SE* = 0.83, *z* = 2.17, p = .03). The standardized beta weight for PDI was (*β* = -0.30, *SE* = 0.07, *z* = -4.41, *p* < .001), the estimated odds ratio showed a decrease of 26% (*Exp* (B) = 0.74, 95% CI [-0.44, -0.17] for Vaccination against COVID-19 for every unit of increase in PDI. ASRS (*β* = 0.024, *SE* = 0.02, *z* = 1.23, *p* = 0.22), STAI-T (*β* = 0.04, *SE* = 0.02, *z* = 1.8, *p* = .07), RAADSN (*β* = 0.09, *SE* = 0.16, *z* = 0.6, *p* = .54),. Psychiatric diagnosis (*β* = -1.18, *SE* = 0.42, *z* = -2.81, *p* < .005), the estimated odds ratio showed a decrease of 69% (*Exp* (B) = 0.31, 95% CI [-2, -0.36] for Vaccination against COVID-19 for every unit of increase in psychiatric diagnosis. Lastly, we added education and sex to Model 4, which showed that the standardized Beta weight for the Constant was (*N* = 256, *β* = 1.51, *SE* = 0.95, *z* = 1.59, p = .11). The standardized beta weight for PDI was (*β* = -0.25, *SE* = 0.07, *z* = -3.49, *p* < .001), the estimated odds ratio showed a decrease of 23% (*Exp* (B) = 0.77, 95% CI [-0.4, -0.11] for Vaccination against COVID-19 for every unit of increase in PDI. ASRS (*β* = 0.016, *SE* = 0.021, *z* = 0.74, *p* = 0.45), STAI-T (*β* = 0.04, *SE* = 0.02, *z* = 2.05, *p* = .04), the estimated odds ratio for STAI-T showed an increase of 4.5% (*Exp* (B) = 1.045, 95% CI [0.003, 0.089] for Vaccination against COVID-19 for every unit of increase in STAI-T. RAADSN (*β* = 0.02, *SE* = 0.16, *z* = 1.26, *p* = .9). Psychiatric diagnosis (*β* = -1, *SE* = 0.45, *z* = -2.20, *p* = .026), the estimated odds ratio for Psychiatric Diagnosis showed a decrease of 64% (*Exp* (B) = 0.36, 95% CI [-1.89, -0.11] for Vaccination against COVID-19 for every unit of increase in Psychiatric Diagnosis. Sex (*β* = -0.57, *SE* = 0.44, *z* = -1.32, *p* = .18), education (*β* = -0.16, *SE* = 0.09, *z* = -1.78, *p* = .07).

**Controlling for Age in Logistic Regression Models**

We also conducted the regression analysis but with only young adults aged between 18-35 (*Supplementary Table* 1) and replicated findings in the main sample adjusting all models for age (*Supplementary Table* 2)

**
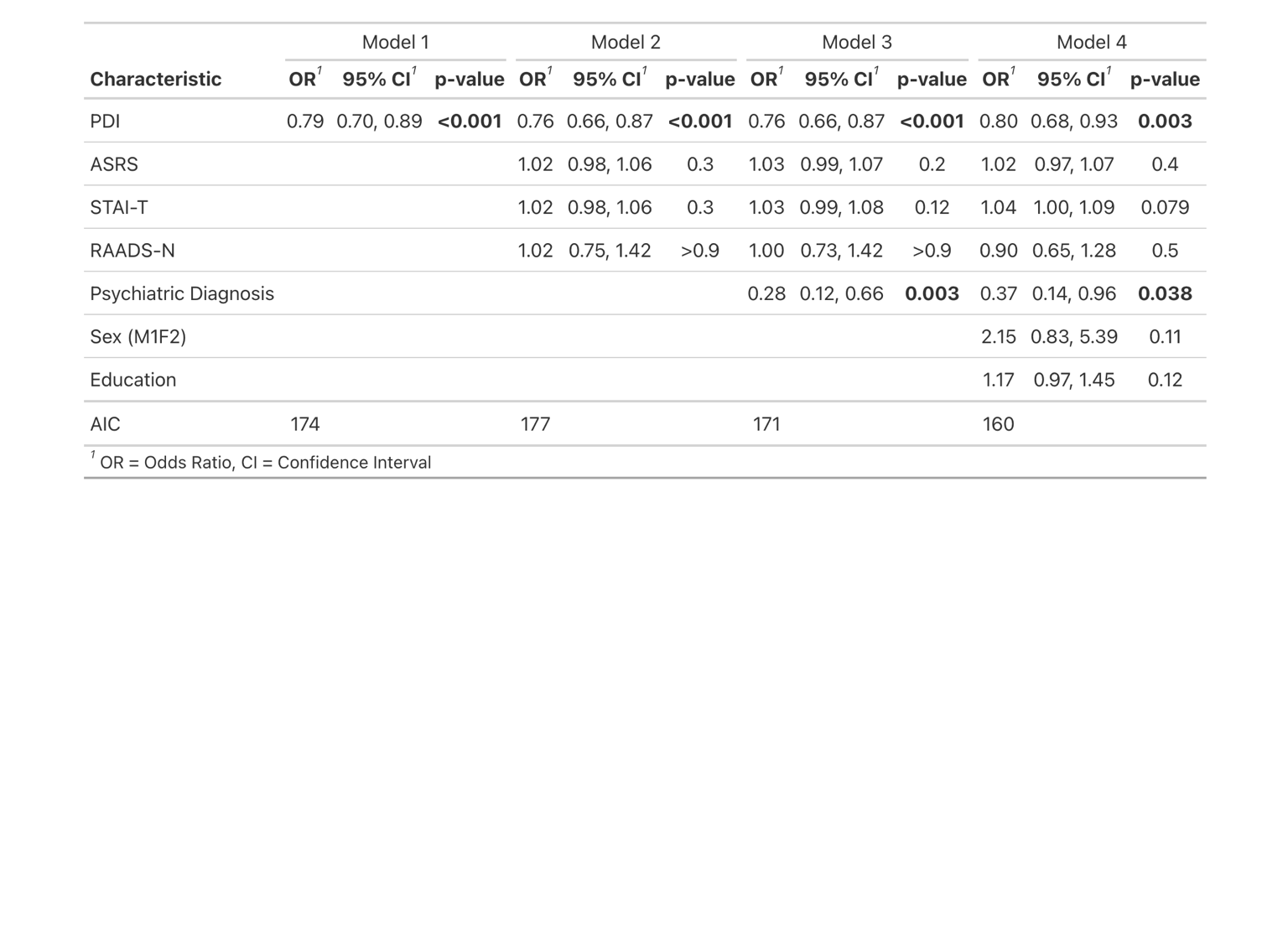
***Supplementary Table 1*. Logistic regression results only including subject between the age of 18-35.

**
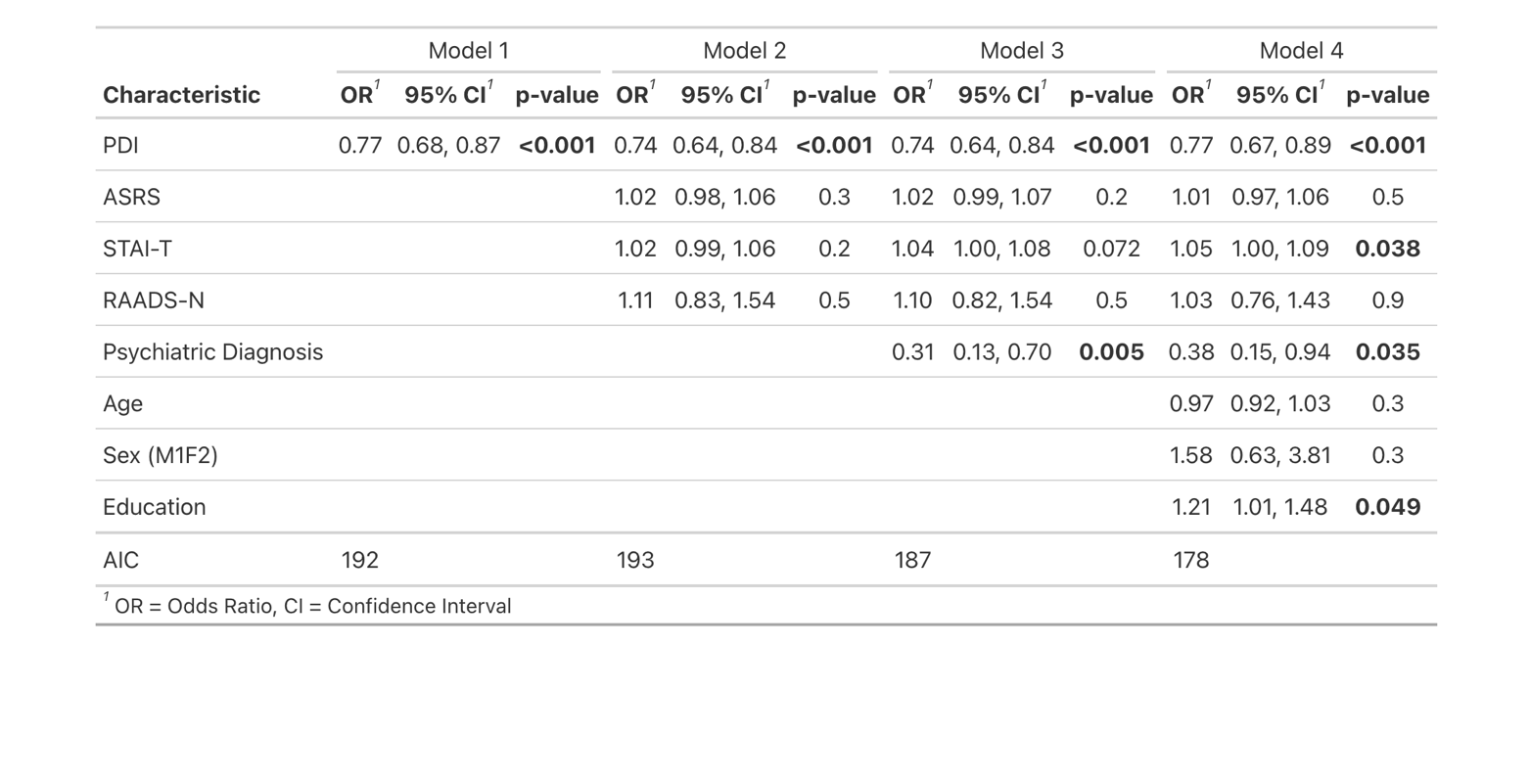
***Supplementary Table 2*. Logistic regression (whole sample) results: all models with adjustments for age.

**Detailed Results of Linear Regression Analyses**

Next, four linear regression analyses were carried out with Time to get vaccinated (in months) as a response variable. We added more predictors for each model in order to control for the effects of covariates. The Model 1 showed that PDI significantly predicts longer time to vaccinate against COVID-19 (*N* = 232, *β* = 0.16, *t* = 3.61, *p* < .001), in the next model we added ASRS, STAI-T and RAADSN as covariates, which showed that PDI still predicts longer time to vaccinate against COVID-19 (*β* = 0.16, *t* = 3.18, *p* < .01), but not ASRS (*β* = 0.002, *t* = 0.012, *p* = .85), STAI-T (*β* = -0.0004, *t* = -0.32, *p* = .97) or RAADSN (*β* = 0.03, *t* = 0.31, *p* = 0.76). Next, we added psychiatric diagnoses as a covariate, showing that PDI predicts longer time to vaccinate (*β* = 0.15, *t* = 3.14, *p* < .01), while the other variables did not; ASRS (*β* = 0.0004, *t* = 0.28, *p* = .78; STAI-T (*β* = 0.001, *t* = 0.10, *p* = .92); RAADSN (*β* = 0.03, *t* = 0.31, *p* = .76); psychiatric diagnosis (*β* = -0.20, *t* = -0.67, *p* = .50). In Model 4, we added sex and education as covariates as well. It showed that PDI predicts longer time to get vaccinated (*β* = 0.13, *t* = 2.69, *p* < .01), sex (*β* = 0.71, *t* = 2.7, *p* < .01), while sex (*β* = -0.67, *t* = -2.22, *p* = .027) and education (*β* = -0.16, *t* = -3.29, *p* < .01) predicted shorter time to get vaccinated. The other variables did not predict shorter or longer time to get vaccinated; ASRS (*β* = 0.001, *t* = 0.11, *p* = .91), STAI-T (*β* = -0.002, *t* = -0.15, *p* = .88), RAADSN (*β* = 0.04, *t* = 0.47, *p* = .65), psychiatric diagnoses (*β* = -0.37, *t* = -1.2, *p* = .23).

**Controlling for Age in Linear Regression Models.**

We also conducted the regression analysis but with only young adults aged between 18-35 (*Supplementary Table* 3) and replicated findings in the main sample adjusting all models for age (*Supplementary Table* 4).

*
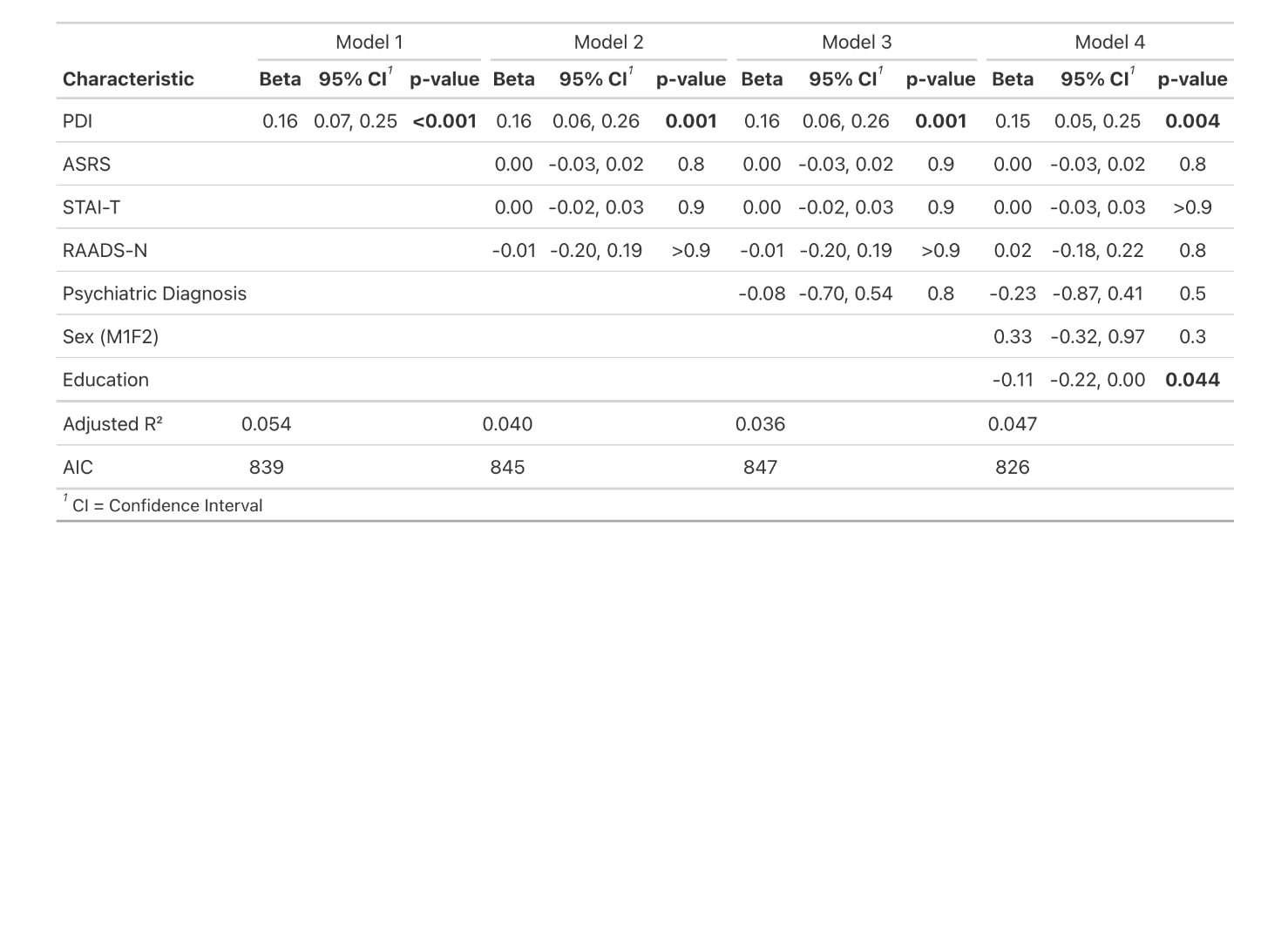
*

***Supplementary Table 3*.** Linear regression results only including subject between the age of 18-35.


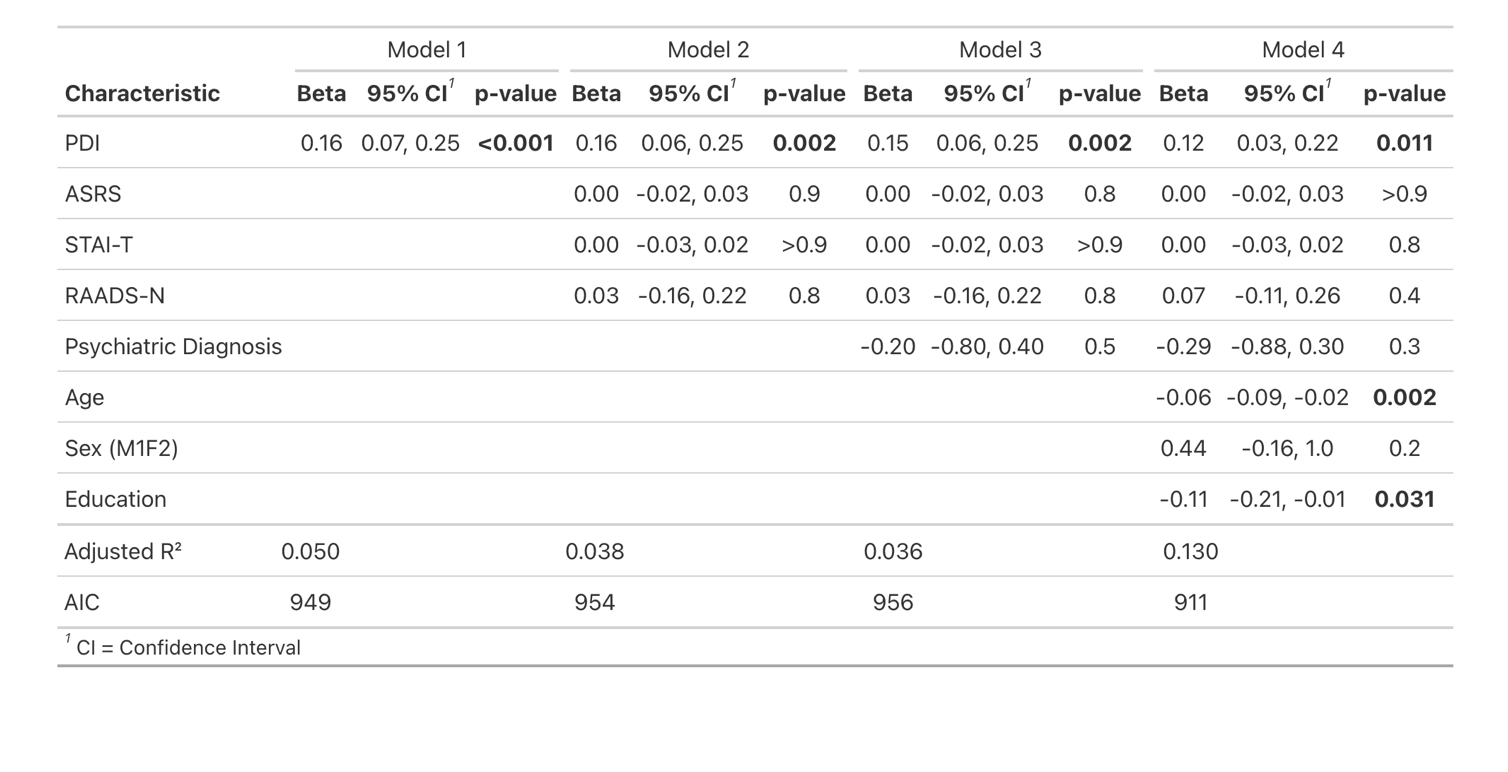
***Supplementary Table 4*.** Linear regression (whole sample) results: all models with adjustments for age.


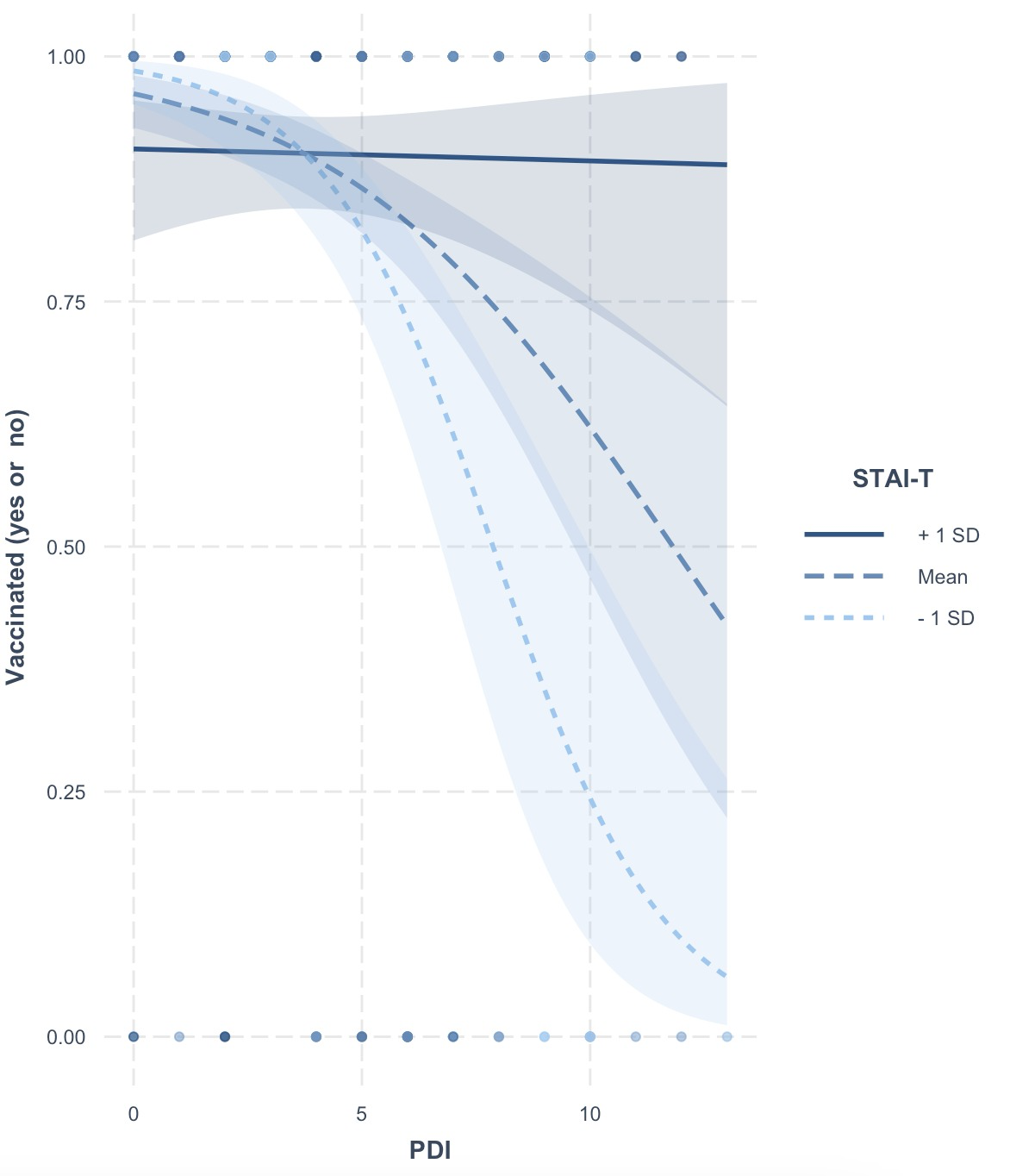


***Supplementary Figure 1*.** Interaction between PDI and STAI-T.

**Detailed Results of Path Analysis**

Results of the path model showed a significant direct effect of PDI to Vaccination (*β* = -0.18, *z* = -3.17, *p* = .002. It also showed a significant contribution of PDI to CCQ Total (*β* = 0.29, *z* = 5.03, *p* < .001), as well as a significant effect from CCQ Total to Vaccination (*β* = -0.34, *z* = -5.96, *p* < .001). The path model showed a partial effect of CCQ Total in mediating the relationship between PDI and decision to get vaccinated (*β* = -0.10, *z* = -3.85, *p* < .001).

**Text analysis.** While both groups were concerned about safety issues, conceptualizations of safety differed between them. We also conducted a group comparison in order to test if the average number of words used, was significantly different between vaccinated and unvaccinated subjects and found that on average, unvaccinated subjects used more words (mean=52.03, SD=83.67, median=20.5, range 2-422) to describe why they did not get vaccinated compared to the vaccinated subjects (average=14.74, SD=10.92, median=11, range 2-61) (W=989.9, p=.002). This result remained significant even after excluding two extreme cases in the non-vaccinated group (W=989.5, p=.009). We then conducted a correlation analysis and found no significant correlation between number of words and PDI (r=-.22, p=0.22) or CCQ (r=-.09, p=0.62). Lastly, we classified every subject’s comment on why they did or did not get vaccinated against COVID-19 into themes (see Figure 3B and Supplement).

**Survey Questions**

1. Have you been offered to get vaccinated against COVID-19?

2. Have you gotten vaccinated against COVID-19?

3. Will you get vaccinate against COVID-19 once you are offered?

4. I have/will vaccinate against COVID-19 because:

- It is important that I get vaccinated to stop the spread of the virus.
- COVID-19 poses a threat to my health
- Jag har familj/släkt eller andra personer i min närhet som är i riskgruppen
- I have family/relatives or other persons close to me that are in the risk group
- Other reasons

If you want to elaborate your answer you can do it below:

5. How long did it take for you to get vaccinated against COVID-19 once you were offered?

- As fast as possible
- A few weeks
- A month
- A few months

If you want to elaborate your answer you can do it below:

6. When did you receive your first vaccination dose? (If you have not had your first dose of vaccine yet, you can skip this question). If you do not remember the exact date, please give an approximate answer (e.g., last two weeks of December 2020).

**References**

Acar, K., Horntvedt, O., Cabrera, A., Olsson, A., Ingvar, M., Lebedev, A.V., and Petrovic, P. (2022). COVID-19 conspiracy ideation is associated with the delusion proneness trait and resistance to update of beliefs. *Sci Rep* 12**,** 10352.

Lebedev, A.V., Acar, K., Garzon, B., Almeida, R., Raback, J., Aberg, A., Martinsson, S., Olsson, A., Louzolo, A., Parnamets, P., Lovden, M., Atlas, L., Ingvar, M., and Petrovic, P. (2021). Psychedelic drug use and schizotypy in young adults. *Sci Rep* 11**,** 15058.

Lebedev, A.V., Acar, K., Horntvedt, O., Cabrera, A.E., Simonsson, O., Osika, W., Ingvar, M., and Petrovic, P. (2023). Alternative beliefs in psychedelic drug users. *Sci Rep* 13**,** 16432.
